# Serum metabolomic profiling facilitates the non-invasive identification of metabolic biomarkers associated with the onset and progression of non-small cell lung cancer

**DOI:** 10.18632/oncotarget.7354

**Published:** 2016-02-12

**Authors:** Leonor Puchades-Carrasco, Eloisa Jantus-Lewintre, Clara Pérez-Rambla, Francisco García-García, Rut Lucas, Silvia Calabuig, Ana Blasco, Joaquín Dopazo, Carlos Camps, Antonio Pineda-Lucena

**Affiliations:** ^1^ Structural Biochemistry Laboratory, Centro de Investigación Príncipe Felipe, Valencia, Spain; ^2^ Molecular Oncology Laboratory, Fundación para la Investigación del Hospital General Universitario, Valencia, Spain; ^3^ Instituto de Investigación Sanitaria La Fe, Hospital Universitario i Politécnico La Fe, Valencia, Spain; ^4^ Computational Genomics Department, Centro de Investigación Príncipe Felipe, Valencia, Spain; ^5^ Department of Medical Oncology, Consorcio Hospital General Universitario, Valencia, Spain; ^6^ Bioinformatics of Rare Diseases (BIER), CIBER de Enfermedades Raras (CIBERER), Valencia, Spain; ^7^ Functional Genomics Node, Instituto Nacional de Bioinformática / Centro de Investigación Príncipe Felipe, Valencia, Spain; ^8^ Department of Medicine, Universitat de València, Valencia, Spain

**Keywords:** NSCLC, metabolomics, biomarkers, early diagnosis, prognosis

## Abstract

Lung cancer (LC) is responsible for most cancer deaths. One of the main factors contributing to the lethality of this disease is the fact that a large proportion of patients are diagnosed at advanced stages when a clinical intervention is unlikely to succeed. In this study, we evaluated the potential of metabolomics by ^1^H-NMR to facilitate the identification of accurate and reliable biomarkers to support the early diagnosis and prognosis of non-small cell lung cancer (NSCLC).

We found that the metabolic profile of NSCLC patients, compared with healthy individuals, is characterized by statistically significant changes in the concentration of 18 metabolites representing different amino acids, organic acids and alcohols, as well as different lipids and molecules involved in lipid metabolism. Furthermore, the analysis of the differences between the metabolic profiles of NSCLC patients at different stages of the disease revealed the existence of 17 metabolites involved in metabolic changes associated with disease progression.

Our results underscore the potential of metabolomics profiling to uncover pathophysiological mechanisms that could be useful to objectively discriminate NSCLC patients from healthy individuals, as well as between different stages of the disease.

## INTRODUCTION

Lung cancer (LC) is the most common cause of cancer death worldwide, accounting for approximately 12% of all cases of cancer, with an incidence of almost two million new cases annually worldwide [1]. The average five-year LC survival rate in early-stage, operable, non-small cell lung cancer (NSCLC) is approximately 50-70%. However, the five-year survival rate drops to 2-5% for patients diagnosed after their tumors have spread distantly [2]. At present, the diagnosis is primarily based on symptoms and detection often occurs at late stages, thus resulting in a very poor prognosis. If the diagnosis could be shifted to early stages, then the overall morbidity for this disease could be dramatically altered.

Recent studies have shown that LC screening using Low Dose Computed Tomography (LDCT) is effective in reducing mortality [3]. However, the large proportion of individuals with indeterminate nodules, the high costs involved and the limited resources available, demand the identification of more accurate risk profiles, ideally based on non-invasive or minimally invasive techniques (i.e., blood, sputum, exhaled air, etc.) [4], in combination with other clinical, epidemiological, imaging, and life-style information. This strategy could be particularly relevant for individuals at-risk for LC as they may have subclinical disease for years before presentation.

Metabolomics, an analytical tool used in combination with pattern recognition approaches, is a very promising approach in systems biology; its objective being the comprehensive analysis of low-molecular weight metabolites in biological samples [5]. It represents a very powerful approach to the understanding of the biological pathways involved in the onset and progression of diseases, providing valuable insights into the molecular mechanisms of pathological processes [6]. The most commonly employed analytical techniques used for metabolic profiling are Nuclear Magnetic Resonance (^1^H-NMR) spectroscopy and Mass Spectrometry (MS). High-resolution ^1^H-NMR spectroscopy provides quantitative analysis of metabolite concentrations and reproducible information with minimal sample handling.

Monitoring specific metabolite levels in serum/plasma, the most commonly used biofluids in clinical metabolomics, has become an important tool for detecting early stages of some oncological diseases [7]. Thus, metabolomics by ^1^H-NMR spectroscopy has been applied for the identification of different biomarkers in renal [8, 9], colorectal [10], pancreatic [11], ovarian [12, 13], and oral cancers [14], as well as in some hematological diseases [15, 16], among others.

In recent years, a number of studies have reported promising results in the characterization of the metabolic profile of LC patients [17-22]. However, a comprehensive approach to the early detection of this disease requires the extensive analysis of a more representative set of samples that could lead to the identification of specific and reliable clinical biomarkers. To that end, in this study, a thorough analysis of the serum metabolic profile of NSCLC patients at different stages of the disease was compared with that corresponding to healthy individuals and patients diagnosed with other benign pulmonary diseases (BPDs). Using a metabolomics approach based on ^1^H-NMR, it was possible to identify and independently validate a set of selective and specific metabolites that could be useful for the early detection of LC in the clinical context. Taken together, the results provide an opportunity for improving current risk stratification models.

## RESULTS

### Non-supervised analysis of the serum samples from the training set reveals that disease status contributes to the metabolic discrimination of healthy individuals and NSCLC patients

Non-supervised analysis of the ^1^H-NMR spectra (Supplementary Figure 1) was carried out to evaluate the potential influence of different clinical variables on the metabolic profiles obtained for the serum samples from the training set. Among all the variables assessed, only classification of the samples according to disease status had an impact in the clustering of the samples (Supplementary Figure 2).

The unsupervised analysis also highlighted the existence of differences between the two independent, training and validation, sets of samples included in the study (data not shown). An analysis of these differences revealed that they were attributed to technical variability, most likely reflecting the existence of differences in the suppression of the residual water signal of the spectra at the time of measurement.

### Supervised analysis of the data reveals the existence of statistically significant differences between the metabolic profiles of NSCLC patients and healthy individuals, as well as between different disease stages

**Figure 1:**
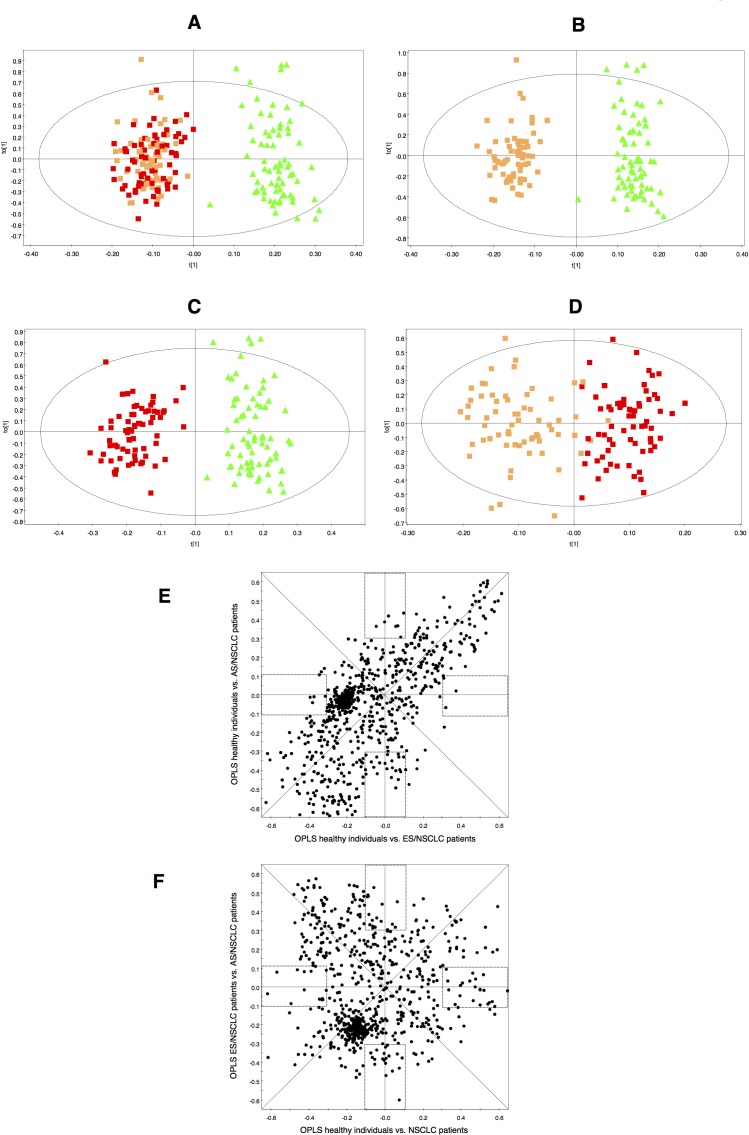
Multivariate modelling resulting from the analysis of serum ^1^H-NMR spectra OPLS-DA score plots for the comparisons between: **A.** healthy individuals (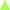) *vs.* NSCLC patients (early-stage and advanced-stage, 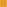 and 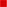, respectively); **B.** healthy individuals (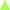) *vs.* early-stage NSCLC patients (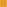); **C.** healthy individuals (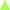) *vs.* advanced-stage NSCLC patients (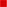) and **D.** early-stage NSCLC patients (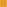) *vs.* advanced-stage NSCLC patients (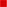). SUS-plots derived from the OPLS-DA models between: **E.** healthy individuals *vs*. early-stage NSCLC patients *(model B, horizontal axis)* and healthy individuals *vs*. advanced-stage NSCLC patients *(model C, vertical axis)*; **F.** healthy individuals *vs*. NSCLC patients *(model A, horizontal axis)* and early-stage NSCLC *vs*. advanced-stage NSCLC patients *(model D, vertical axis)*. Rectangles indicate unique biomarkers for each model.

Discriminant statistical models (OPLS-DA) were built based on the comparisons between the different groups of samples included in the training set (Figure 1). This analysis revealed that serum samples from NSCLC patients, compared with healthy individuals, exhibit a specific serum metabolic profile (R^2^ = 0.931; Q^2^ = 0.873) characterized by statistically significant differences in the concentrations of a number of metabolites (Figure 1A). A similar analysis performed to compare the serum metabolomics profile of NSCLC patients at early and advanced stages of the disease (R^2^ = 0.779; Q^2^ = 0.592) showed that disease progression has also a reflection in the metabolic profile of patients (Figure 1D).

An analysis of the metabolic differences based on the results of the corresponding shared and unique structures plots (SUS-plots) (Figure 1E,1F) revealed that the most significant variations between the serum metabolic profile of NSCLC patients and the healthy individuals were shared regardless of the stage of the disease (Figure 1A,1B,1C), and that they were different from those found between early and advanced stages of NSCLC (Figure 1D).

### Specific combinations of metabolites are involved in the discrimination between healthy individuals and NSCLC patients, and between different stages of NSCLC

An inspection of the contribution to the separation between groups resulted in the identification of the spectral signals, and eventually the metabolites, that contributed more to the discrimination between the groups of samples being compared. Using this approach, a total of 18 metabolites showed statistically significant differences when comparing the serum metabolite levels of NSCLC patients and healthy individuals (Table 1A, Supplementary Figure 3A), and 17 when comparing NSCLC patients at early and advanced stages of the disease (Table 1B, Supplementary Figure 3B).

**Table 1A:** Mean intensities and variations of the most significant metabolites found in the comparison between healthy individuals and NSCLC patients

Metabolite	δ ^1^H (ppm)^a^	Mean spectral intensity ± s.e.m. (arbitrary units)		% variation	*P*-value^b^
		Healthy Group	NSCLC patients		
**HDL (CH_3_)**	0.85-0.79	55.14 ± 1.50	45.55 ± 0.73	−17.39	0.0000*
VLDL (CH_3_)	0.91-0.85	66.10 ± 1.49	65.17 ± 0.78	−1.40	0.9070
**Leucine/Isoleucine**	0.97-0.91	14.40 ± 0.25	16.67 ± 0.26	15.75	0.0000*
3-hydroxybutyrate	1.20-1.18	8.22 ± 0.15	8.60 ± 0.20	4.59	0.8585
**LDL/VLDL (CH_2_)_n_**	1.31-1.20	229.06 ± 6.10	197.77 ± 3.26	−13.66	0.0001*
**Unknown 1**	1.40-1.36	4.01 ± 0.11	5.04 ± 0.14	25.65	0.0000*
**Adipic acid**	1.60-1.52	15.97 ± 0.69	13.79 ± 0.34	−13.64	0.0406*
**Acetate**	1.91-1.90	1.29 ± 0.04	1.56 ± 0.04	21.22	0.0001*
**Lipids (CH_2_-C=C)**	2.02-1.93	40.15 ± 0.46	35.56 ± 0.31	−11.44	0.0000*
**N-Acetyl-cysteine**	2.05-2.02	23.50 ± 0.32	27.01 ± 0.44	14.94	0.0000*
Lipids (CH_2_-CO)	2.26-2.19	14.19 ± 0.65	13.17 ± 0.30	−7.15	0.5775
**Glutamate**	2.36-2.33	2.14 ± 0.07	2.95 ± 0.07	37.65	0.0000*
**Glutamine**	2.47-2.41	5.82 ± 0.19	4.98 ± 0.11	−14.37	0.0002*
**Choline–N(CH_3_)_3_^+^**	3.21-3.18	23.56 ± 0.67	17.60 ± 0.34	−25.30	0.0000*
**Methanol**	3.36-3.35	1.01 ± 0.05	1.81 ± 0.05	78.81	0.0000*
**Glycerol**	3.80-3.78	2.90 ± 0.09	3.63 ± 0.08	25.16	0.0000*
**Creatine**	3.92-3.91	0.90 ± 0.04	1.20 ± 0.03	33.82	0.0000*
**Lactate**	4.13-4.08	10.45 ± 0.38	13.74 ± 0.42	31.53	0.0000*
**Threonine**	4.30-4.21	6.92 ± 0.12	5.86 ± 0.09	−15.34	0.0000*
Glucose	5.24-5.21	11.92 ± 0.35	12.63 ± 0.26	5.92	0.2202
**Lipids (CH=CH)**	5.37-5.24	32.92 ± 0.75	28.08 ± 0.45	−14.70	0.0000*
**Histidine**	7.78-7.74	0.37 ± 0.01	0.29 ± 0.01	−20.76	0.0000*

a Chemical shift region used for quantification

b *P*-value calculated using the Mann–Whitney-Wilcoxon test

* Statistically significant (*P* < 0.05)

**Table 1B:** Mean intensities and variations of the most significant metabolites found in the comparison between earlystage and advanced-stage NSCLC patients

Metabolites	δ ^1^H (ppm)^a^	Mean spectral intensity ± s.e.m. (arbitrary units)		% variation	*P*-value^b^
		ES/NSCLC	AS/NSCLC		
HDL (CH_3_)	0.85-0.79	45.72 ± 1.08	45.70±1.01	−0.06	0.9054
VLDL (CH_3_)	0.91-0.85	65.66 ± 0.94	64.71 ± 1.23	−1.43	0.8602
**Leucine/Isoleucine**	0.97-0.91	15.89 ± 0.34	17.41 ±0.37	9.59	0.0039*
**3-hydroxybutyrate**	1.20-1.18	9.38 ± 0.31	7.85 ± 0.21	−16.26	0.0000*
LDL/VLDL (CH_2_)_n_	1.31-1.20	203.92 ± 4.48	191.89 ± 4.65	−5.90	0.1151
**Adipic acid**	1.60-1.52	14.61 ± 0.50	13.00 ± 0.44	−11.01	0.0248*
**Lysine**	1.75-1.65	9.15 ± 0.26	10.36 ± 0.26	13.16	0.0025*
Lipids (CH_2_-C=C)	2.02-1.93	35.27 ± 0.40	35.84 ±0.48	1.60	0.1403
**N-Acetyl-cysteine**	2.05-2.02	24.51 ± 0.50	29.39 ± 0.59	19.90	0.0000*
**Glutamate**	2.09-2.05	10.09 ± 0.24	12.30 ± 0.27	21.86	0.0000*
**Lipids (CH_2_-CO)**	2.26-2.19	14.55 ± 0.42	11.85 ± 0.37	−18.54	0.0000*
**Glutamine**	2.47-2.41	5.91 ± 0.17	5.03 ± 0.15	−14.84	0.0005*
**Citrate**	2.96-2.64	4.66 ± 0.11	5.41 ± 0.11	16.26	0.0000*
Choline–N(CH_3_)_3_^+^	3.21-3.18	18.05 ± 0.50	17.17 ± 0.46	−4.86	0.2081
Unknown 2	3.55-3.54	3.22 ± 0.09	3.40 ± 0.09	5.76	0.1852
**Unknown 3**	3.58-3.56	4.98 ± 0.17	5.97 ± 0.16	19.71	0.0000*
**Valine**	3.61-3.59	3.55 ± 0.10	4.30 ± 0.10	21.05	0.0000*
**Glycerol**	3.67-3.63	13.49 ± 0.38	15.76 ± 0.38	16.82	0.0001*
**Creatine**	3.92-3.91	1.25 ± 0.04	1.43 ± 0.04	14.43	0.0031*
**H α/β amino acids**	4.02-3.92	8.86 ± 0.26	10.54 ± 0.27	18.92	0.0000*
**Lactate**	4.13-4.08	13.01 ± 0.52	14.59 ± 0.64	12.15	0.0274*
**Glucose**	5.24-5.21	13.06 ± 0.37	11.86 ± 0.37	−9.19	0.0058*
Lipids (CH=CH)	5.37-5.24	28.89 ± 0.58	27.51 ± 0.70	−4.79	0.2948
**Phenylalanine**	7.43-7.40	0.31 ± 0.02	0.47 ± 0.03	52.10	0.0000*

a Chemical shift region used for quantification

b *P*-value calculated using the Mann–Whitney-Wilcoxon test

* Statistically significant (*P* < 0.05)

### A logistic regression analysis of the data identifies a minimal set of metabolites involved in the discrimination of NSCLC patients and healthy individuals

A logistic regression equation was obtained based on the analysis of the metabolites exhibiting significant statistical differences between both groups (Table 1A). Using this approach, characteristic higher levels of lactate and methanol and lower levels of glutamine, choline and threonine were found in serum samples from NSCLC patients compared with healthy individuals (Table 2). Internal validation of the logistic regression equation was performed by evaluating the AUC values of the ROC curves for each individual metabolite (Supplementary Figure 4).

**Table 2:** Characteristics of the logistic regression equation obtained for the discrimination between healthy individuals and NSCLC patients

Metabolite	β^a^	OR^b^	1/OR	*P*-value
Glutamine	−1.70	0.18	5.47	0.0032*
Choline	−0.41	0.66	1.51	0.0011*
Methanol	4.60	99.63	0.01	0.0001*
Lactate	0.34	1.41	0.71	0.0347*
Threonine	−1.82	0.16	6.19	0.0036*
**Constant**	17.70	4.89E+07	2.05E-08	0.0032*

a β: Coefficient of logistic regression

b OR: odds ratio

* Statistically significant (*P* < 0.05)

### External validation of the predictive ability of the OPLS-DA and the logistic regression models

Samples included in the validation set were used to assess the predictive ability of the orthogonal projection to latent structures discriminant analysis (OPLS-DA) and the logistic regression models based on the training set. To remove the variation between the NMR data obtained for the two sets of samples, standardization of NMR signal intensities for the training and the validation sets of samples was achieved using the *ComBat* method [23].

**Figure 2:**
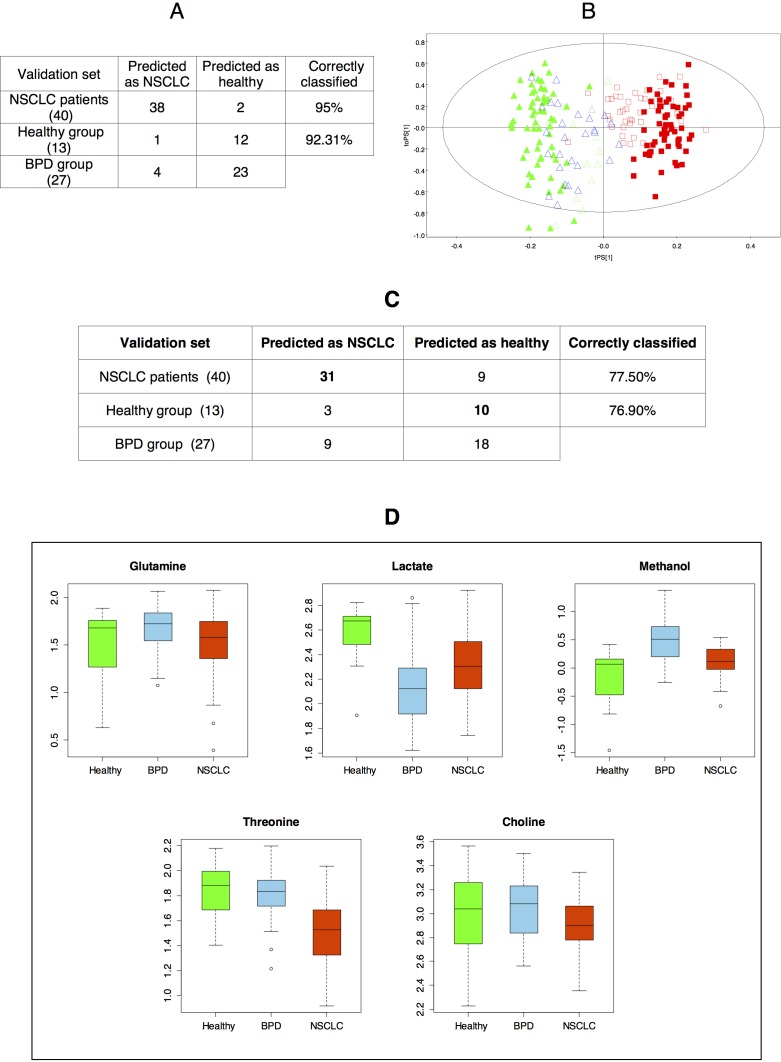
**A.** Prediction results derived from the OPLS-DA model corresponding to the comparison between healthy individuals and NSCLC patients (training set). **B.** OPLS-DA score plot displaying the prediction of the samples included in the validation set based on the model corresponding to the training set (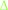: healthy individuals -validation set-; 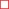: NSCLC patients -validation set-; 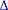: BPD patients -validation set-; 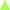: healthy individuals -training set-; 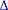: NSCLC patients -training set-). **C.** Misclassification table based on the logistic regression equation. **D.** Boxplot (log scale) representing the intensities of the metabolites included in the logistic regression equation for the different groups. For each box, the central line is the median, the edges of the box are the upper and lower quartiles, the whiskers extend the box by a further ±1.5 interquartile range (IQR), and outliers are plotted as individual points.

Thus, it was found that, based on the OPLS-DA model obtained for the training set, 95% of patients diagnosed with NSCLC, as well as all but one of the healthy individuals included in the validation set, were correctly classified. An evaluation of the behavior of the serum samples obtained from patients diagnosed with BPDs was also carried out. In this case, it was found that 23 out of the 27 BPD samples (85.2%) were classified as healthy individuals. Overall, the multivariate statistical model obtained for the discrimination between NSCLC patients and healthy individuals was 95% specific and 92.31% sensitive (87.50% for all non-cancer samples) (Figure 2A, 2B).

The probability of belonging to the group of NSCLC patients for the samples included in the validation set was also evaluated using the logistic regression equation. Unsurprisingly, the ability of the prediction based on the logistic regression model was lower (Figure 2C) than that based on the OPLS-DA multivariate statistical model, since the latter includes information about all the regions of the spectra. Overall, 77.3% of samples in the validation set were correctly classified; 77.5% of the NSCLC patients and 76.9% of samples of the healthy individuals included in the validation set were correctly classified (70% for all non-cancer samples).

The decrease in the percentage of BPD samples classified as healthy individuals (85.2% (OPLS-DA model) *versus* 66.6% (logistic regression model)) was further investigated by evaluating the levels of the five metabolites from the logistic regression equation in the samples from the validation set (Figure 2D). This analysis revealed that patients diagnosed with BPDs, compared with healthy individuals, exhibit statistically significant higher levels of methanol. These results prompted the analysis of the potential differences existing between patients diagnosed with BPDs and healthy volunteers or NSCLC patients to get a better understanding of the metabolic changes that were specific of NSCLC patients and those ones shared with BPDs.

### Patients diagnosed with BPDs have a metabolic profile different from both healthy individuals and NSCLC patients

OLPS-DA statistical models were thus generated to compare the metabolic profiles of patients diagnosed with BPDs and NSCLC patients (R^2^ = 0.972; Q^2^ = 0.856) or healthy individuals (R^2^ = 0.963; Q^2^ = 0.782) (Supplementary Figure 5A,5B). The analysis of the contribution coefficients of each spectral region in these two statistical models, together with the SUS-plots obtained for the comparison of the three OPLS-DA models (Supplementary Figure 5A,5B,5C) allowed the identification of the shared and unique metabolic differences relevant in each statistical model (Supplementary Figure 5D, 5E, 5F). This analysis revealed that the serum metabolic profile of BPD patients is characterized by statistically significant higher levels of methanol and lower levels of lactate compared with healthy individuals, and statistically significant higher levels of methanol, choline and LDL/VLDL and lower levels of lactate and glucose compared with NSCLC patients (Table 3).

**Table 3:** Variations of the mean intensities of the most relevant metabolites involved in the discrimination between BPD patients and healthy individuals or NSCLC patients

Metabolites	δ^1^H (ppm)^a^	BPD *vs*. Healthy		BPD *vs*. NSCLC	
		% variation	*P*-value^b^	% variation	*P*-value^b^
HDL (CH_3_)	0.85-0.79	−6.55	0.3602	−9.37	0.1073
**VLDL (CH_3_)**	0.91-0.85	−0.19	0.7539	−16.47	0.0002*
**Leucine/Isoleucine**	0.97-0.91	2.52	0.9319	8.54	0.0360*
3-hydroxybutyrate	1.20-1.18	−2.98	0.5490	20.64	0.0735
**LDL/VLDL (CH_2_)_n_**	1.31-1.20	1.61	0.9319	−23.28	0.0000*
Unknown 1	1.40-1.36	34.21	0.1424	−7.80	0.0676
**Adipic acid**	1.60-1.52	4.30	0.8867	−28.57	0.0001*
**Acetate**	1.91-1.90	−15.53	0.7979	6.96	0.0078*
**Lipids (CH_2_-C=C)**	2.02-1.93	−6.28	0.1424	−15.00	0.0000*
**N-Acetyl-cysteine**	2.05-2.02	−8.02	0.0392*	−6.80	0.0152*
Lipids (CH_2_-CO)	2.26-2.19	4.63	0.8867	−12.33	0.1674
**Glutamate**	2.36-2.33	−7.84	0.2175	43.35	0.0000*
**Glutamine**	2.47-2.41	−15.34	0.1065	−14.08	0.0136*
**Lipids (CH=CH-CH2-CH=CH)**	2.79-2.68	−0.06	0.6077	−15.40	0.0000*
**Choline –N(CH_3_)_3_^+^**	3.21-3.18	−3.46	0.8197	−14.45	0.0461*
**Proline**	3.30-3.34	−27.10	0.0130*	−15.46	0.0434*
**Methanol**	3.36-3.35	−45.45	0.0010*	−34.12	0.0002*
**Unknown 2**	3.55-3.54	−10.03	0.3166	17.93	0.0091*
Unknown 3	3.58-3.56	−13.86	0.2765	9.10	0.1714
Valine	3.61-3.59	−11.64	0.2068	9.14	0.2150
**Glycerol**	3.80-3.78	−7.20	0.4406	17.94	0.0027*
**Creatine**	3.92-3.91	−12.39	0.2175	22.52	0.0005*
**Creatinine**	4.04-4.03	−0.24	0.6898	−24.18	0.0000*
**Myo-inositol**	4.07-4.05	10.28	0.4578	−33.58	0.0000*
**Lactate**	4.13-4.08	49.44	0.0002*	16.92	0.0136*
**Threonine**	4.30-4.21	4.85	0.5878	−26.09	0.0000*
**Glucose**	5.24-5.21	−5.59	0.2639	42.78	0.0000*
**Lipids (CH=CH)**	5.37-5.24	2.23	0.3602	−21.01	0.0000*

a Chemical shift region used for quantification

b *P*-value calculated using the Mann–Whitney U test

* Statistically significant (*P* < 0.05)

## DISCUSSION

Efforts to identify NSCLC biomarkers that could help to better understand disease pathogenesis and to effectively identify patients at early stages of the disease remain a fundamental goal in this area [24]. In this context, our study represents the first attempt, based on the analysis of a significant number of samples, to characterize and compare the specific serum metabolic profile of NSCLC patients at different stages of the disease with those of healthy individuals and patients diagnosed with different BPDs.

The presence of lower levels of high-density lipoprotein / low-density lipoprotein / very-low-density lipoprotein (HDL/LDL/VLDL) in NSCLC patients correlates well with the relationship between decreased serum lipid levels and the development of some oncological processes [25]. Variations in the lipid levels in oncological patients have been previously associated with an increased uptake of cholesterol, an essential component of cell membranes, by tumor cells [16]. Changes in lipid metabolism could also explain the variations in the concentration of adipic acid, a metabolite that is associated with abnormalities in the metabolism of fatty acids, in patients at different stages of the disease [26]. The lower level of serum choline, a precursor of membrane phospholipids, observed in the group of patients with NSCLC could also be associated with the increased demand of this metabolite by tumor cells due to the their high proliferation rate [27, 28]. Serum metabolic profile of NSCLC patients is also characterized by significantly higher levels of lactate and lower levels of glucose. These results are consistent with the increased uptake of glucose and its conversion to lactate described in various tumor tissues [29, 30], a phenomenon associated with the well-known Warburg effect [31].

Previous studies have reported significant alterations in the serum amino acid profile of cancer patients, most probably reflecting the hypermetabolic state and increased demand of amino acids during tumor development [32-34]. Our data show that NSCLC patients, compared with healthy individuals, exhibit higher serum levels of leucine/isoleucine (15.75%), N-acetyl-cysteine (14.94%) and glutamate (37.65%), and lower levels of glutamine (−14.37%), threonine (−15.34%) and histidine (−20.76%). Increased concentrations of leucine/isoleucine, N-acetyl-cysteine and glutamate and decreased concentrations of glutamine are also observed when the serum metabolic profiles of NSCLC patients at early and advanced stages of the disease are compared. The specific decrease of serum threonine and histidine levels observed in NSCLC patients, compared with healthy individuals, most probably reflects the up-regulation of the glycine/serine/threonine and pyrimidine metabolic pathways, respectively, that have been described as metabolic hallmarks of NSCLC tumor-initiating cells [35]. Furthermore, disease progression is characterized by a specific increase in the serum concentrations of lysine (13.16%), valine (21.05%) and phenylalanine (52.10%). The significant increase in the serum concentration of phenylalanine is in agreement with the down-regulation of gene modules involved in phenylalanine metabolism observed in tissue samples from NSCLC patients [36], and could reflect a limited ability of lung cancer cells to process this amino acid at advanced stages of the disease. The decrease in serum glutamine levels in NSCLC patients is consistent with other cancer studies [11, 37, 38] where it has been associated with increased metabolic activity derived from the conditions of hypoxia and hypermetabolism observed in the tumor environment [39]. A recent study has also revealed the important role that glutamine, as a nitrogen source for the synthesis of nucleotides and amino acids, plays in these conditions [40]. In this context, the hydrolysis of glutamine for the production of ammonia and glutamate to balance the pH in tumor cells could explain the high serum levels of glutamate observed in the group of NSCLC patients. Interestingly, the sustained increase in N-acetyl-cysteine levels suggest that metabolic pathways leading to the production of antioxidant species are up-regulated in NSCLC patients. This finding provides further support to a recent study conducted in a genetically engineered mouse model that mimics early human NSCLC [41]. In this study, authors concluded that antioxidants play an important role in LC progression by reducing the expression of p53, a key tumor suppressor protein.

The increase in creatine levels deserves special attention as a chemically related metabolite, creatine riboside, was recently associated [42] with NSCLC in a urinary metabolomics study. This metabolite was found to be elevated in the urine of NSCLC patients and associated with poor prognosis. Creatine is transformed into phosphocreatine, an energy reservoir, by creatine kinase isoenzyme BB, an enzyme that has been shown to exhibit high serum levels in NSCLC patients [43, 44]. Therefore, the observation of elevated levels of creatine in NSCLC patients compared with healthy individuals, as well as between different disease stages, could be associated with the high metabolic activity of this neoplastic process.

Finally, our metabolomics study reveals that there are significant statistical differences between the serum metabolic profile of patients diagnosed with BPDs and healthy individuals (R^2^ = 0.963; Q^2^ = 0.782) or NSCLC patients (R^2^ = 0.972; Q^2^ = 0.856). Our results are partially in agreement with a recent NMR metabolomics study carried out using serum samples from NSCLC and chronic obstructive pulmonary disease (COPD) patients [20]. Thus, Deja *et al*. reported that the serum metabolic profile of NSCLC patients, compared with patients diagnosed with COPD, was characterized by higher levels of lactate and lower levels of methanol [42]. Our results also show that the serum metabolic profile of BPD patients, compared with NSCLC patients, is effectively characterized by higher levels of methanol and lower levels of lactate. In contrast, they observed higher levels of choline in serum samples from NSCLC patients, and we report lower levels of choline in serum of NSCLC patients, our results being in agreement with previous results obtained from the analysis of tissue samples from LC tumors [45].

Overall, our results show that NSCLC patients, compared with healthy individuals and patients diagnosed with BPDs, exhibit characteristic serum metabolic profiles, and that disease stage has also a significant impact in the serum metabolic profile of patients. A specific combination of five metabolites, based on a logistic regression analysis, is presented, enabling the discrimination between healthy individuals, BPD patients and NSCLC patients with a 77.5% specificity and a 76.9% sensitivity (70% for all non-cancer samples). The combination of all the metabolites involved in the discrimination between healthy individuals and NSCLC patients should also be explored as they provide a specific signature, both in terms of magnitude and change direction, of the metabolic alterations responsible for the onset/progression of NSCLC with a 95% specificity and 92.31% sensitivity (87.50% for all non-cancer samples). The strategy described in this work provides a sensitive, specific, and minimally invasive method that may aid in the early diagnosis and staging of NSCLC and the optimization of current risk stratification models.

## MATERIALS AND METHODS

### Patient cohorts

A total of 296 serum samples were analyzed by ^1^H-NMR (Table 4). Samples from NSCLC patients were classified into two groups [46]:

**Table 4:** Clinical and demographic characteristics of the individuals included in the study

Total number	Training data set			Validation set			
	Healthy	ES/NSCLC	AS/NSCLC	Healthy	BPD	ES/NSCLC	AS/NSCLC
	74	72	70	13	27	20	20
**Gender**							
Female	13	8	12	2	13	5	7
Male	61	64	58	11	14	15	13
**Age ± s.e.m**^a^	56 ± 1.55	63 ± 1.17	63 ± 1.29	47 ± 1.78	52 ± 2.88	68 ± 1.48	61 ± 2.28
**Smoking habits**							
Ex-smoker	22	25	21	1	8	9	10
Smoker	31	42	20	8	7	8	4
Non-smoker	21	5	5	3	11	3	6
Unknown			24	1	1		
**Histology**							
Adenocarcinoma		24	32			9	16
Large-cell carcinoma		2	4			1	1
Squamous-cell carcinoma		38	27			9	
Other or unspecified		8	7			1	3
**Stage**							
IA		10				5	
IB		27				4	
IIA		1				5	
IIB		17				4	
IIIA		17				2	
IIIB			18				
IV			52				20
**Other pathology**							
COPD					9		
TBC					3		
Pneumonia					4		
CB					2		
Other					9		

Abbreviations: ES/NSCLC, early-stage non-small cell lung cancer; AS/NSCLC, advanced-stage non-small cell lung cancer; BPD, benign pulmonary diseases; COPD, chronic obstructive pulmonary disease; TBC, tuberculosis; CB, chronic bronchitis.

a Age=mean years at time of sample collection ± s.e.m (standard error of mean).

- Advanced stage NSCLC: Patients diagnosed with advanced NSCLC (stage IIIB with pleural effusion or stage IV, non-squamous histologies) with no other concomitant malignancies [47, 48]. Samples were obtained prior to chemotherapy.
- Early stage NSCLC: Newly diagnosed patients with resectable NSCLC (stage IA-IIIA) without prior chemotherapy. A pre-surgery serum sample was collected from each patient.

Furthermore, the study included two control groups: 87 serum samples from healthy individuals without any acute or chronic inflammatory conditions, and a group 27 serum samples from patients diagnosed with BPDs in the validation set.

Patient recruitment and sampling procedures were performed in accordance with the Declaration of Helsinki and applicable local regulatory requirements and laws and after approval from the Ethics Committees of all participating institutions.

### Sample preparation and ^1^H-NMR acquisition

Serum samples were immediately stored at −80°C after collection. At the time of NMR analysis, samples were thawed on ice. 300 μL of 10% D_2_O buffer (5 mM TSP, 140 mM Na_2_HPO_4_, 0.04% NaN_3_, pH 7.4) were added to 300 μL of serum. ^1^H-NMR spectra were acquired using a Bruker Avance II 600 MHz spectrometer equipped with triple resonance cryo-probe with a cooled ^13^C preamplifier (TCI) at 310 K (37°C) [49, 50]. Metabolites of interest were identified using Amix v 3.9.7 in combination with the Bruker NMR Metabolic Profiling Database BBIOREFCODE 2.0.0 database (Bruker Biospin, Rheinstetten, Germany), as well as other existing public databases and literature reports [12, 22, 51]. NMR experiments for each set were independently acquired at two different times.

### Multivariate statistical analysis

^1^H-NMR spectra were binned using Amix 3.9.7 (Bruker Biospin, Rheinstetten, Germany) over the region δ 9.02-0.14 ppm. The water (δ 5.06-4.30 ppm) and urea signal (δ 5.85-5.60 ppm) regions were excluded from the analysis to avoid interference arising from differences in water suppression and variability from urea signal, respectively. All bucket intensities were normalized to the total area of the corresponding spectra. Bucket tables generated were imported into SIMCA-P 12.0 software (Umetrics AB, Sweden). Prior to statistical analysis, data were Pareto scaled. The *ComBat* method, included in the “sva” R package [52], was applied to compensate differences due to batch effects.

PCA was used to examine the intrinsic variability within the data set, to observe clustering or separation trends and for the identification of outliers. OPLS-DA was applied to minimize the possible contribution of inter-group variability and to further improve separation between the groups of samples. The default method of 7-fold internal cross validation was applied, from which Q^2^Y (predictive ability parameter, estimated by cross-validation) and R^2^Y (goodness of fit parameter) values were extracted. Those parameters, together with the corresponding permutation tests (*n* = 100), were used for the evaluation of the quality of the OPLS-DA models obtained. SUS-plots were also obtained to evaluate the shared (metabolites aligned with the diagonals) and unique differences (metabolites aligned with the axes) found when comparing two OPLS-DA statistical models.

### Quantitative analysis of selected metabolites

The main metabolites contributing to group discrimination in each model were integrated using Amix 3.9.7. Normality in variable distribution was assessed using the Kolmogorov-Smirnov test. Statistical significance was assessed using the Mann-Whitney U test. A *P*-value < 0.05 (confidence level 95%) was considered statistically significant.

### Logistic regression

Logistic regression analysis was performed using the “stats” R package [53]. Univariate logistic regression was carried out with the *Introduction* method, and the *Forward stepwise regression* method was used for the multivariate logistic regression. Odds ratio (OR) values were calculated for all the variables included in the equation. A *P*-value < 0.05 (confidence level 95%) was considered statistically significant.

## SUPPLEMENTARY MATERIAL FIGURES


