# Supplementary material for: Serum metabolomic profiling facilitates the non-invasive identification of metabolic biomarkers associated with the onset and progression of non-small cell lung cancer
Source: Oncotarget. 2016 Feb 12;7(11):12904–16. doi: 10.18632/oncotarget.7354 (PMC4914330; doi:10.18632/oncotarget.7354)
Supplement: Supplementary file 1 [file oncotarget-07-12904-s001.pdf]

Serum metabolomic profiling facilitates the non-invasive identification of metabolic biomarkers associated with the onset and progression of non-small cell lung cancer

Supplementary Material

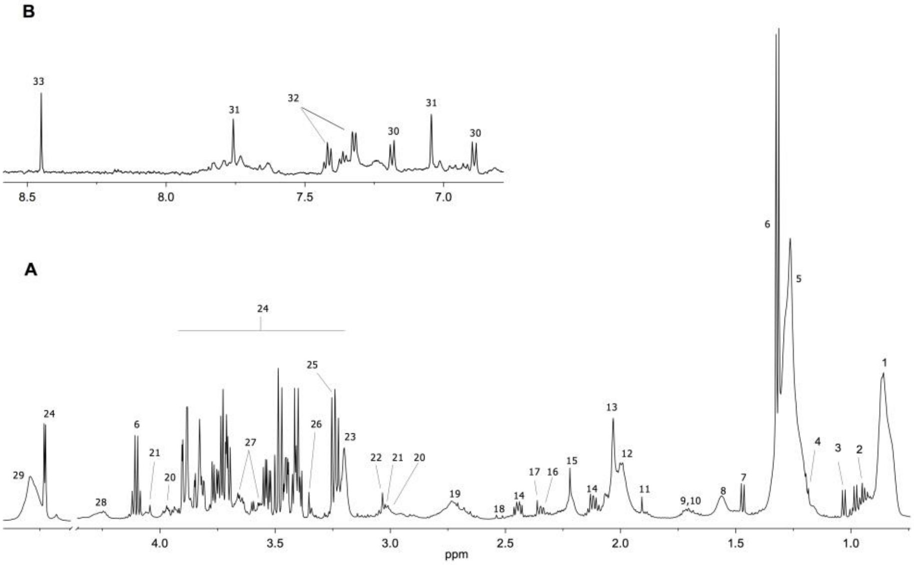

Supp. **Figure 1. Assignment of the ^{1}H-NMR signals of a representative 600 MHz one-dimensional Carr–Purcell–Meiboom–Gill (1D-CPMG) ^{1}H-NMR spectrum of an early-stage NSCLC patient serum sample measured at 310K**. **A**, full spectrum (d=5.25–0.75 ppm) and **B**, magnification of aromatic region (d=8.60–6.75 ppm). Peak assignments: 1, lipids (–CH_3) (mainly LDL/VLDL); 2, leucine/isoleucine; 3, valine; 4, 3-hydroxybutyrate; 5, lipids $(CH_2)_n$(mainly LDL/VLDL); 6, lactate; 7, alanine; 8, adipic acid; 9, lysine; 10, arginine; 11, acetate; 12, lipids (CH_2–C=C); 13, NAC (acetyl signals from glycoproteins); 14, glutamine; 15, lipids (CH_2–CO); 16, glutamate; 17, pyruvate; 18, citrate; 19, lipids (CH=CH–CH_2–CH=CH–); 20, creatine; 21, creatinine; 22, acetate; 23, choline; 24, glucose; 25, trimethylamine N-oxide; 26, methanol; 27, glycerol; 28, threonine; 29, lipids (–CH=CH–); 30, tyrosine; 31, histidine; 32, phenylalanine; 33, formate.

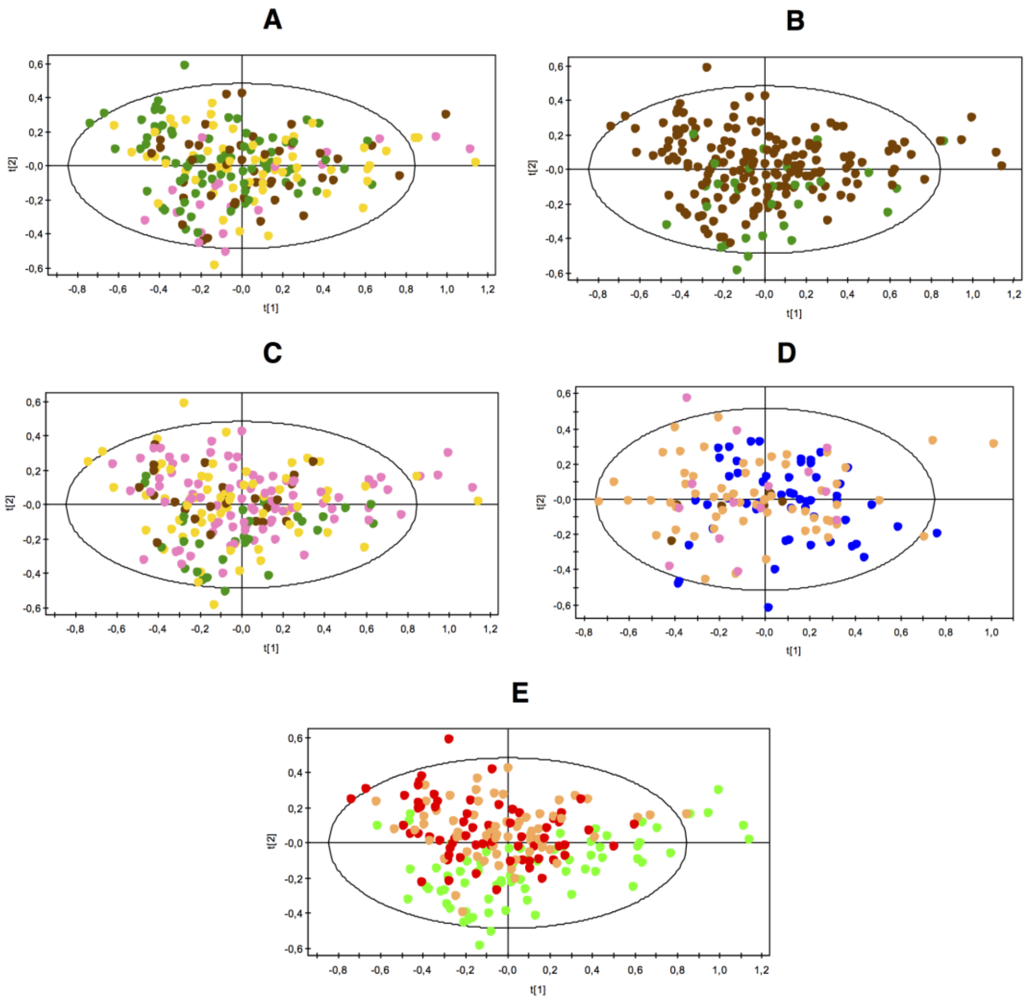

Supp. Figure 2. Principal Component Analysis (PCA) score plots corresponding to the samples included in the training set. Samples are classified according to: (**A**) Age: ●: <45; ●: 45-55; ●: 55-65; ●: >65; (**B**) Gender: ●: male; ●:female; (**C**) Smoking habits: ●: Non-smoker; ●: Ex-smoker; ●: Smoker; ●: Unknown; (**D**) Histology: ● :Adenocarcinoma; ●:Squamous-cell carcinoma; ●:Large-cell carcinoma ●:Other or unspecified; and (**E**) Group: ●: ES/NSCLC; ●: AS/NSCLC; ●: Healthy individuals.

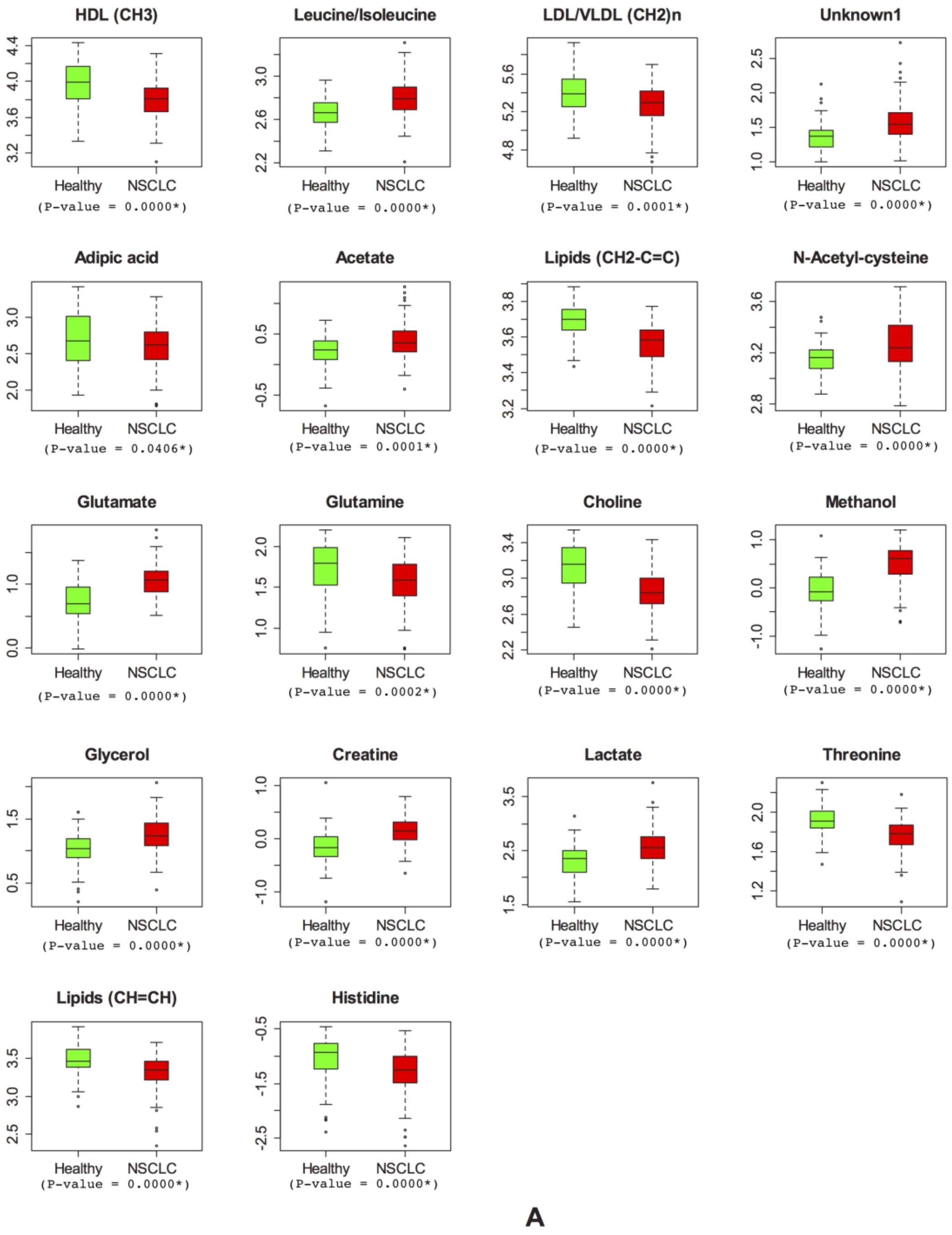

Supp. Figure 3A. **Boxplot (log scale) for the most significant metabolites found in the comparison between healthy individuals and NSCLC patients**. For each box, the central line is the median, the edges of the box are the upper and lower quartiles, the whiskers extend the box by a further ±1.5 interquartile range (IQR), and outliers are plotted as individual points. Statistically significant *P*-values (*P*-value<0.05) are indicated with asteriks (*).

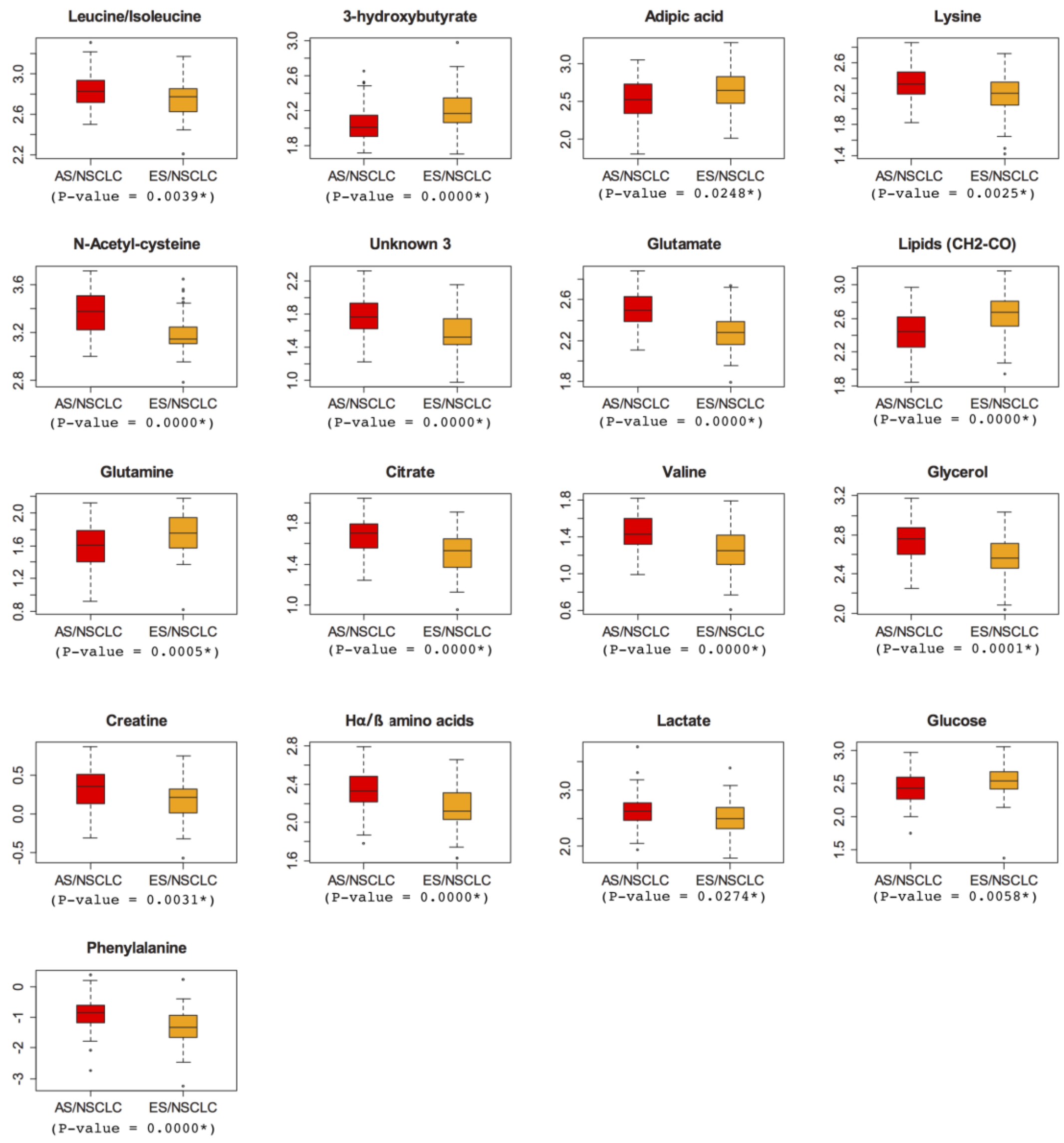

B

Supp. Figure 3B. Boxplot (log scale) for the most significant metabolites found in the comparison between early-stage (ES) and advanced-stage (AS) NSCLC patients. For each box, the central line is the median, the edges of the box are the upper and lower quartiles, the whiskers extend the box by a further ±1.5 interquartile range (IQR), and outliers are plotted as individual points. Statistically significant *P*-values (*P*-value<0.05) are indicated with asteriks (*).

	AUC	95% CI[a]		Cut-off point	Sensitivity	Specificity
		Lower	Upper			
**Methanol**	0,82	0,74	0,90	1,55	68%	90%
**Lactate**	0,79	0,72	0,88	10,71	70%	82%
**Glutamine**	0,65	0,56	0,75	5,63	75%	55%
**Choline -N(CH$_3$)$_3^+$**	0,82	0,76	0,90	20,30	82%	73%
**Threonine**	0,75	0,67	0,84	5,71	80%	67%

[a]Asymptotic 95% confidence interval, lower and upper bound

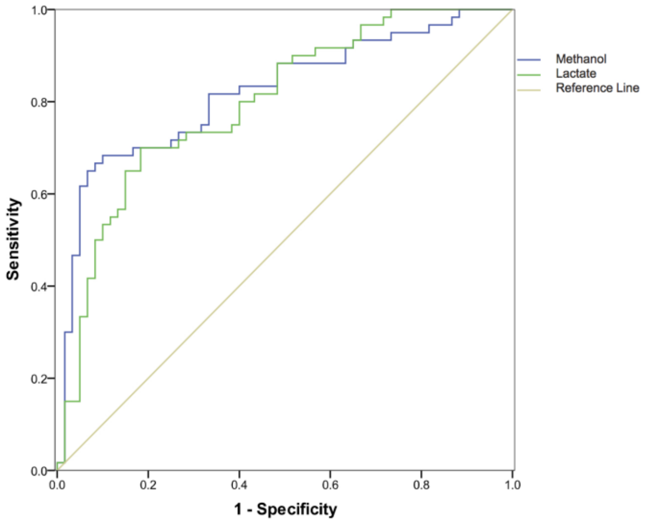

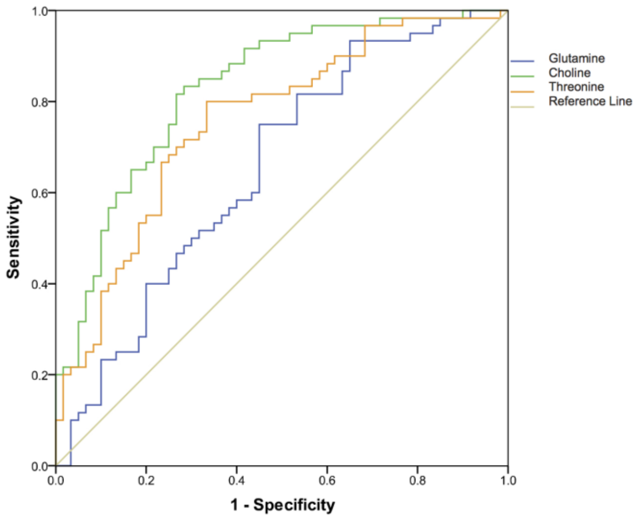

Supp. Figure 4. Internal validation of the logistic regression equation.

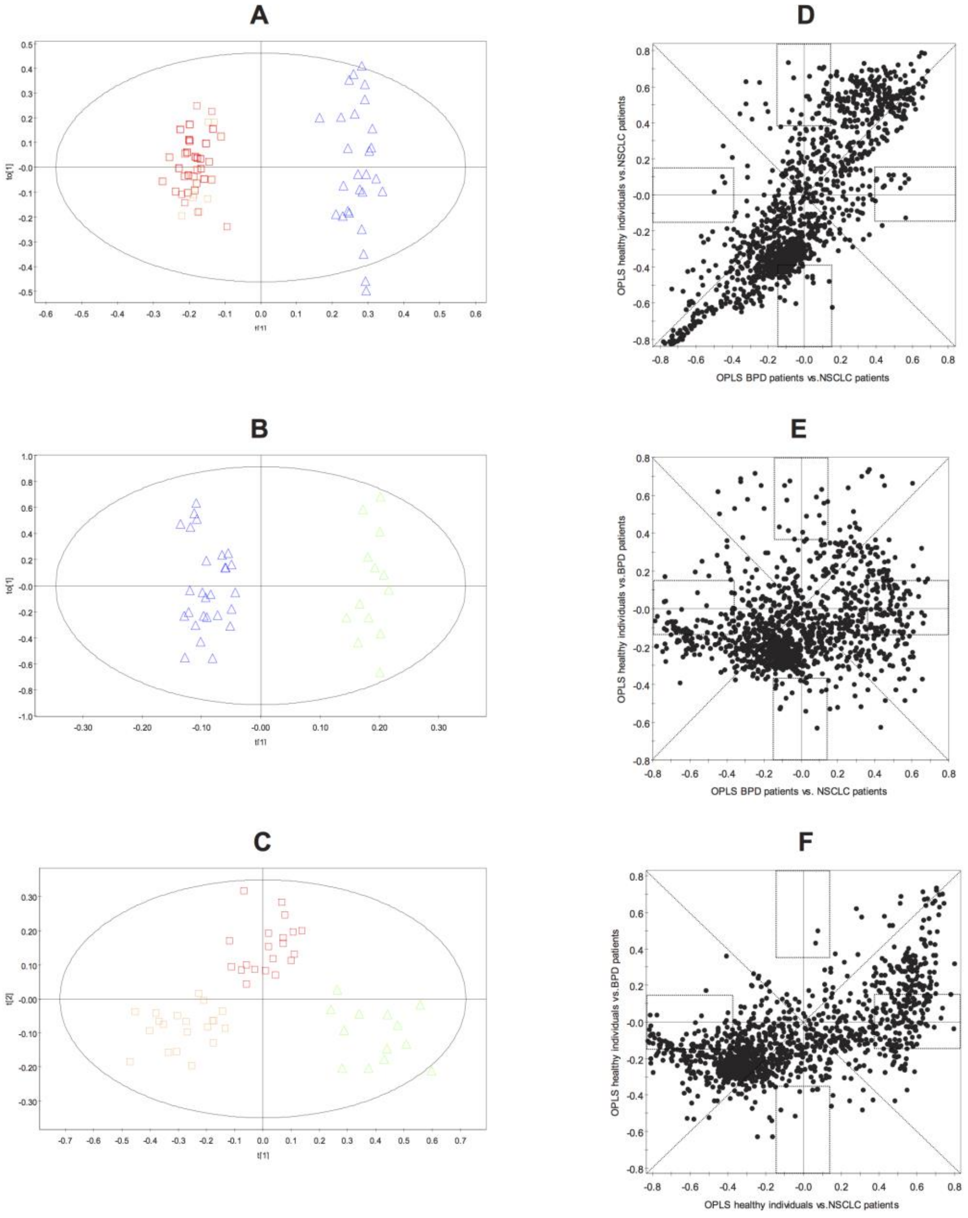

Supp. Figure 5. **Multivariate modelling resulting from the analysis of serum ^{1}H-NMR spectra from the validation set**. OPLS-DA score plots for the comparisons between: (**A**) BPD (Δ) *vs.* NSCLC patients (early-stage and advanced-stage, □ and □, respectively); (**B**) healthy individuals (Δ) *vs.* BPD patients (Δ), and (**C**) healthy individuals (Δ) *vs.* NSCLC patients (early-stage and advanced-stage, □ and □, respectively). SUS-plots derived from the OPLS-DA models between: **(D)** BPD *vs*. NSCLC patients *(model A, horizontal axis)* and healthy individuals *vs.*

NSCLC patients *(model C, vertical axis)*; **(E)** BPD *vs*. NSCLC patients *(model A, horizontal axis)* and healthy individuals *vs*. BPD patients *(model B, vertical axis)* and (**F**) Healthy individuals *vs.* NSCLC patients (*model C, horizontal axis*) and healthy individuals *vs.* BPD patients (*model B, vertical axis*). Rectangles indicate unique biomarkers for each model.
